# Neuronal differentiation of hair-follicle-bulge-derived stem cells co-cultured with mouse cochlear modiolus explants

**DOI:** 10.1371/journal.pone.0187183

**Published:** 2017-10-30

**Authors:** Timo Schomann, Laura Mezzanotte, John C. M. J. De Groot, Marcelo N. Rivolta, Sanne H. Hendriks, Johan H. M. Frijns, Margriet A. Huisman

**Affiliations:** 1 Department of Otorhinolaryngology and Head & Neck Surgery, Leiden University Medical Center, Leiden, South Holland, the Netherlands; 2 Optical Molecular Imaging Group, Department of Radiology, Erasmus Medical Center, Rotterdam, South Holland, the Netherlands; 3 Centre for Stem Cell Biology, Department of Biomedical Science, University of Sheffield, Sheffield, England, United Kingdom; University of Colorado Boulder, UNITED STATES

## Abstract

Stem-cell-based repair of auditory neurons may represent an attractive therapeutic option to restore sensorineural hearing loss. Hair-follicle-bulge-derived stem cells (HFBSCs) are promising candidates for this type of therapy, because they (1) have migratory properties, enabling migration after transplantation, (2) can differentiate into sensory neurons and glial cells, and (3) can easily be harvested in relatively high numbers. However, HFBSCs have never been used for this purpose. We hypothesized that HFBSCs can be used for cell-based repair of the auditory nerve and we have examined their migration and incorporation into cochlear modiolus explants and their subsequent differentiation. Modiolus explants obtained from adult wild-type mice were cultured in the presence of EF1α-copGFP-transduced HFBSCs, constitutively expressing copepod green fluorescent protein (copGFP). Also, modiolus explants without hair cells were co-cultured with DCX-copGFP-transduced HFBSCs, which demonstrate copGFP upon doublecortin expression during neuronal differentiation. Velocity of HFBSC migration towards modiolus explants was calculated, and after two weeks, co-cultures were fixed and processed for immunohistochemical staining. EF1α-copGFP HFBSC migration velocity was fast: 80.5 ± 6.1 μm/h. After arrival in the explant, the cells formed a fascicular pattern and changed their phenotype into an ATOH1-positive neuronal cell type. DCX-copGFP HFBSCs became green-fluorescent after integration into the explants, confirming neuronal differentiation of the cells. These results show that HFBSC-derived neuronal progenitors are migratory and can integrate into cochlear modiolus explants, while adapting their phenotype depending on this micro-environment. Thus, HFBSCs show potential to be employed in cell-based therapies for auditory nerve repair.

## Introduction

Sensorineural hearing loss (SNHL) can be induced by a variety of causes, such as genetic mutations, prolonged exposure to loud noise, ototoxic drug treatment, or simply as a result of ageing, and is frequently the result of irreversible damage to, and subsequent loss of, hair cells and auditory neurons [1]. The degree of neuron loss is of significance for hearing-impaired patients using a cochlear implant (CI), because this device directly stimulates auditory neurons. Stem cells may counteract the effect of neuronal degeneration and the ensuing neuron loss [2]. Here, stem cells may serve different purposes: their descendants can replace spiral ganglion cells and/or glial cells, but regeneration may also be stimulated by stem-cell-driven paracrine secretion of cytokines and growth factors [3]. This could be of interest in clinical applications, because it can be surmised that in deaf patients stem cells will lack trophic support from hair cells and perhaps also from non-sensory cells.

In 2012, Chen *et al*. reported that human embryonic stem-cell-derived otic progenitors could be successfully incorporated into inner ear tissue in an animal model of SNHL and differentiated into neurons which subsequently innervated the hair cells [4]. Nevertheless, it would be ideal for future clinical applications to have suitable autologous stem cells in order to avoid the risk of graft rejection. Cell-based therapy using autologous neural-crest-derived stem cells (NCSCs) may represent such an attractive therapeutic option. During embryogenesis, neural crest cells delaminate from the dorsal neural tube and migrate laterally towards the otic placode, where they develop in close association with cells from the otic placode [5–7]. Moreover, during embryogenesis, the neural crest and the otic placode develop and differentiate in a similar way, especially since both originate from the lateral border of the neural plate [5, 6, 8–13]. Hence, NCSCs may easily be directed towards an appropriate neuronal or glial phenotype, which is necessary for auditory nerve repair [5]. Another attractive characteristic of NCSCs is that they are migratory by nature. Given this property, it may be surmised that stem cells implanted into the basal cochlear turn will move from the transplantation site further into the damaged area, or will migrate to more apically located cochlear turns. In the adult body, niches with residing NCSCs, such as the hair follicle bulge, can be easily accessed. Only minimally invasive surgery is necessary to harvest hair-follicle-bulge-derived stem cells (HFBSCs), which opens the way to autologous transplantations. Taken together, HFBSCs would be well suited to study cell-based therapy in animal models of SNHL. However, HFBSCs have never been used in inner ear regeneration research. Therefore, before starting transplantation studies, it is important that a number of prerequisites are met *in vitro*. The current study aimed on establishing if HFBSCs demonstrate three key factors to ensure successful auditory nerve repair in CI users: (1) retention of the nature as a migrating neural crest cell, (2) their incorporation into damaged cochlear tissue (i.e., without hair cells) and (3) their capability to differentiate into auditory neuronal or glial precursor phenotypes after incorporation–without the support of growth factors from hair cells.

To mimic the situation in hearing-impaired patients, we used cochlear modiolus explants–devoid of sensory hair cells–from adult mice and co-cultured the explants with genetically manipulated HFBSCs to investigate the aforementioned key factors [14]. We used stem cells which express copepod green fluorescent protein (copGFP) under the control of either the constitutively active elongation factor 1α (EF1α) or the promotor of doublecortin (DCX), the neuronal migration protein [15].

## Material and methods

### Animals

Healthy, adult (> 23 days) male and female mice (strain C57Bl/6) were bred and housed in the Animal Care Facility of Leiden University Medical Center (LUMC, the Netherlands). Animal care and handling were in accordance with the guidelines and regulations as stipulated by the Dutch Experiments on Animals Act (WoD) and the European Directive on the Protection of Animals Used for Scientific Purposes (2010/63/EU) and approved by the Animal Experiments Committee of the LUMC (DEC permit 10172).

### Culture and transduction of HFBSCs

Mouse whisker pads were taken out within 10 minutes after euthanasia by cervical dislocation and stored in DMEM/Ham’s F-12 (Biochrom AG, Berlin, Germany) containing 1% Glutamax (100x; Gibco, Bleiswijk, the Netherlands) and 1% antibiotic/antimycotic solution (100x; Sigma-Aldrich, St. Louis, MO, USA). Hair follicles were dissected from the whisker pads and the bulge region was excised as previously described [16, 17]. Each hair follicle bulge was placed in one well of a 12-well plate (TPP Techno Plastic Products AG, Trasadingen, Austria) pre-coated with an aqueous poly-D-lysine (PDL) solution (0.01 mg/ml; Sigma-Aldrich) and cultured in basic growth medium (BGM) until an outgrowth of 200–400 cells was reached. BGM consists of DMEM/Ham's F-12 1:1 (Biochrom AG, Berlin, Germany), 1% GlutaMax™ (100x; Gibco, Bleiswijk, the Netherlands) and 1% antibiotic/antimycotic solution supplemented with 10% fetal bovine serum (FBS; Gibco), 2% B-27® supplement without vitamin A (50x; Gibco), 1% N-2 MAX media supplement (100x; R&D Systems™, Minneapolis, MN, USA), recombinant human basic fibroblast growth factor (20 ng/ml; R&D Systems), and recombinant human epidermal growth factor (20 ng/ml; R&D Systems). The cultures were maintained in a humidified incubator at 37°C and 5% CO_2_. Cells were enzymatically detached, counted, pooled and seeded at a density of 2.5x10^3^ cells/cm^2^ in PDL-coated dishes. At a confluence of 60%-70%, the cultures were passaged according to the procedure mentioned above.^13,14^ At a yield of 4x10^5^ cells, usually after 4–5 passages, HFBSCs were frozen in 10% DMSO in FBS and stored at -80°C until use. After thawing, HFBSCs were cultured in BGM on PDL-coated dishes until 80% confluence and subsequently transduced with a third-generation lentiviral vector. To monitor HFBSCs, the lentiviral vector pCDH-EF1α-Luc2-T2A-copGFP (EF1α-copGFP) [18], containing the constitutively-active elongation factor-1α (EF1α) promoter and a T2A-linking sequence, was used. The T2A-linking sequence results in equimolar expression of firefly luciferase (Luc2) and copepod green fluorescent protein (copGFP). In addition, HFBSCs were transduced with the lentiviral vector pCDH-DCX-Fer-P2A-Luc2-T2A-copGFP (DCX-copGFP) [15], Vector production and transduction procedures have been described in detail previously [11]. HFBSCs were seeded in PDL-coated 12-well plates at a cell density of 2.5x10^4^ cells/well and maintained in a humidified incubator at 37°C and 5% CO_2_. After attachment, the cells were transduced using a MOI (multiplicity of infection) of 10 as previously described with an efficiency of 83.6 ± 8.2% (mean ± SEM) [14]. Vector production and HFBSC transduction were performed under appropriate biosafety level conditions (ML-II) in accordance with the National Biosafety Guidelines and Regulations for Research on Genetically Modified Organisms. Procedures and protocols were reviewed and approved by the LUMC Biosafety Committee (GMO permit 08–129). Transduced cells were cryopreserved in FBS containing 10% DMSO until used.

### Isolation and dissection of adult mouse modiolus and quarter-turn explants

Auditory bullae were dissected from the adult mouse skull (>23 days) within 10 minutes after euthanasia of the mice and the cochleas were removed and placed in sterile DMEM/Ham's F-12 1:1 containing 1% GlutaMax™ (100x) and 1% antibiotic/antimycotic solution supplemented with 10% FBS. The bony capsule of the cochlea was gently peeled off, the lateral wall tissue was discarded and the whole modiolus taken out using a dissection microscope. The modiolus with the organ of Corti still attached was placed in sterile BGM in a humidified incubator until further processing (within two hours).

Some of the specimens were directly co-cultured with HFBSCs in order to investigate the migration of transduced HFBSCs towards whole modioli. Another portion of the specimens was used to prepare quarter-turn explants. These were prepared by gently cutting the isolated modiolus along a transverse plane and removing the central core of auditory neurons and any excess bone from the inner rim of the osseous spiral lamina followed by dissection, resulting in whole-turn explants containing the osseous spiral lamina and the organ of Corti. Next, the explants were cut into four and these quarter-turn explants were placed in PDL-coated wells.

### Degeneration of hair cells

**Fig 1:**
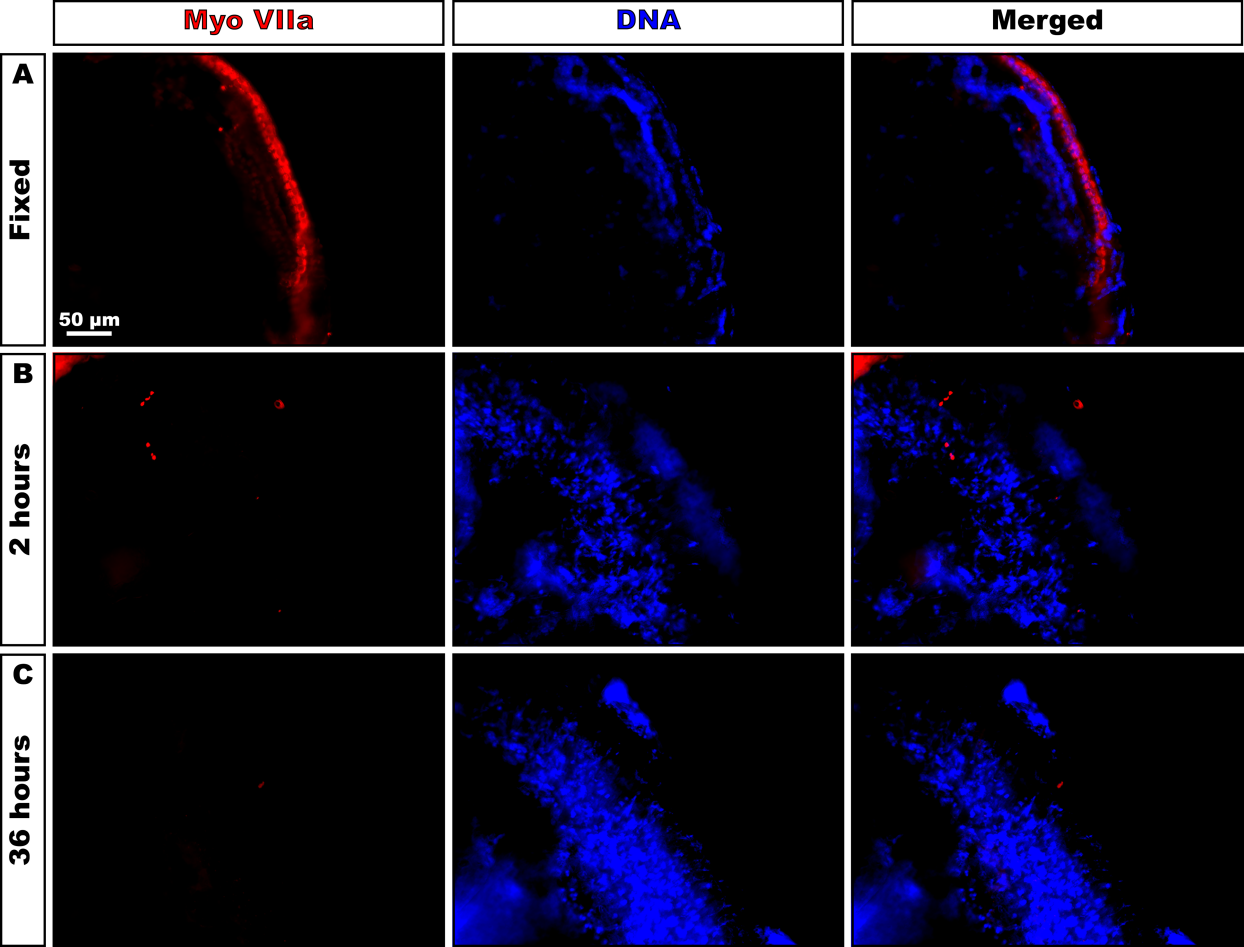
Degeneration of hair cells in cultured explants. (A) Staining for myosin VIIa shows the presence of hair cells in cochleas immediately fixed in formaldehyde prior to microdissection (upper lane; Fixed). (B-C) Quarter-turn explants stored in BGM for 2 hours (B) or 36 hours (C) prior to formaldehyde fixation do not stain for the myosin VIIa, indicating that all hair cells have been lost during culturing.

To mimic a sensory epithelium devoid of hair cells, comparable to the damaged epithelium in deaf patients, we had to ascertain the absence of hair cells under our culture conditions. Therefore, instead of culturing the explants in artificial perilymph, the modioli were kept in BGM for two hours or cultured in PDL-coated dishes for 36 hours and subsequently fixed for immunohistochemistry (Fig 1).

### Migration of HFBSCs

**Fig 2:**
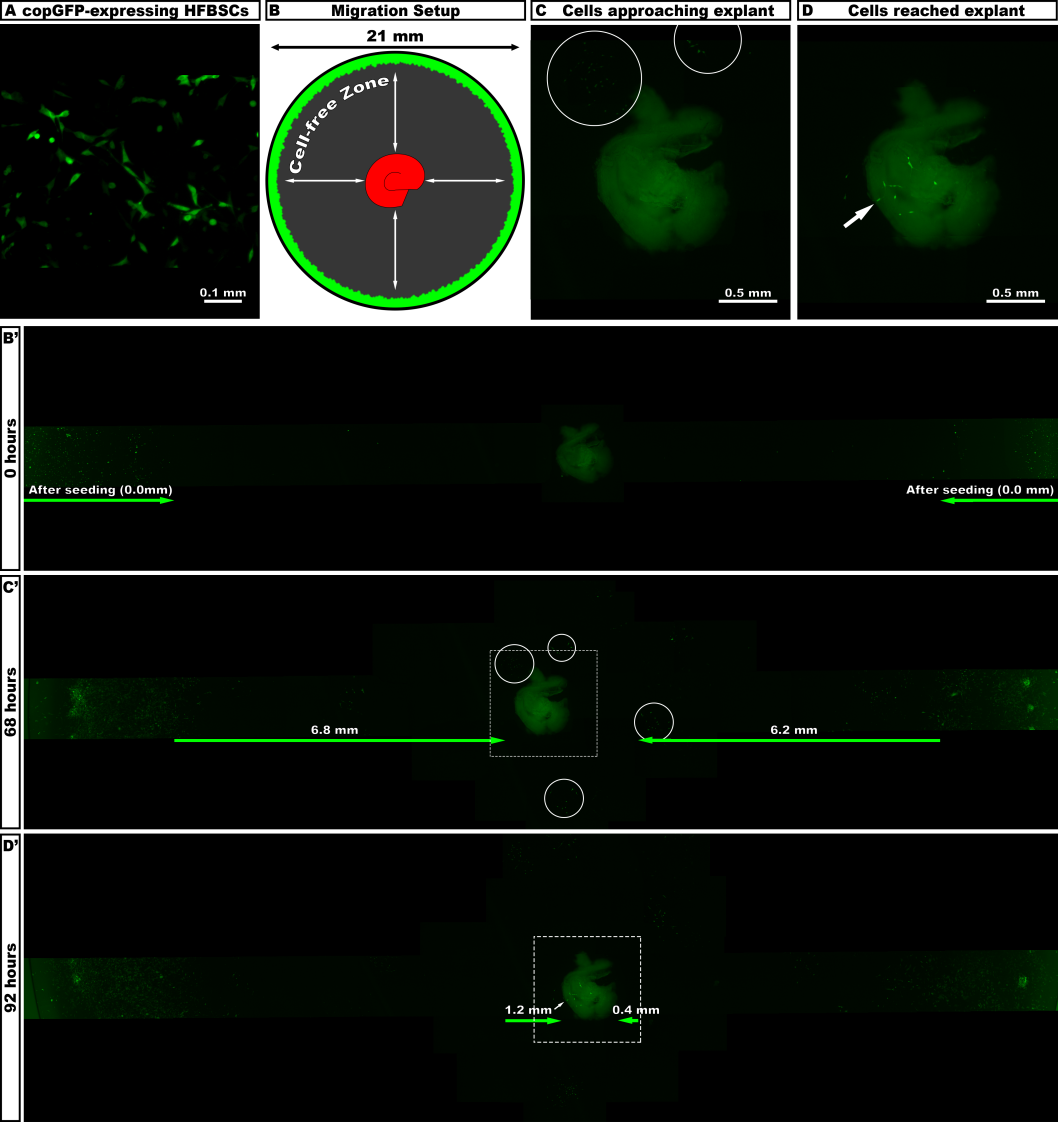
Migration of copGFP-expressing cells. (A) Green fluorescent HFBSCs before co-culture expressed copGFP. (B) Schematic set-up of the migration experiment. At the border of the well (bottom diameter 21 mm), cells were seeded (green) and a modiolus was placed in the middle. (B’) Light-microscope image illustrating the situation directly after seeding and addition of medium (0 h). Fluorescent cells are located at the border of the well, while the modiolus is located in the center as indicated by faint background fluorescence. (C) Groups of cells approached the modiolus (circles) after 68 hours, as is demonstrated at high magnification. (C’) Overview of area depicted in C (boxed area) reveals that brightly fluorescent HFBSCs radially migrated up to 6.8 mm and 6.2mm, respectively, from their point of origin (i.e., the border of the well) towards the modiolus. (D) After 96 hours, HFBSCs reached the modiolus. The arrow indicates the position of the cells located underneath the modiolus. (D’) Overview of area depicted in D (boxed area) illustrating how the first cells reached the modiolus after migrating another 1.2 mm and 0.4 mm, respectively (arrow), after 92 hours. Cells migrated over a cell-free zone (gray) towards the modiolus. In control experiments, i.e. wells without a modiolus in the center, cells proliferated at the border of the well, but did not migrate towards the center of the well (data not shown).

The migratory potential of EF1α-copGFP-transduced HFBSCs (Fig 2A) was investigated by seeding the cells alongside the border of a well in a PDL-coated 12-well dish, while a whole modiolus explant was placed in the center (for a schematic set-up, see Fig 2B). Cells and modiolus explant were allowed to attach at 37°C for 60 minutes, after which 500 μl BGM were added carefully (Fig 2B’). Cell migration velocity was calculated by dividing the migration distance by the time (hours) at various time points (Fig 2B’–2D’). Cell migration distance (in mm) was the distance between the border of the area occupied by the cells after seeding and the position of the cells towards the brim of the explant. Measurements continued until the first fluorescent cells were visible underneath the modiolus explant (Fig 2D). Migration was monitored using an Olympus IX70 fluorescence microscope (FITC filter settings) equipped with a Leica DFC340 FX digital camera. Digital images were acquired and stored using Leica Application Suite Advanced Fluorescence (LAS AF) software (version 1.9.0). All images were subsequently processed using Adobe® Photoshop® CC software (version 2014.2.1).

### Co-culture of HFBSCs

To start the co-culture experiment, 10 μl BGM containing 2x10^4^ HFBSCs were pipetted on top of quarter-turn explants. The co-cultures were incubated at 37°C for 60 minutes, allowing attachment of both cells and explants to the bottom of the PDL-coated well, after which 500 μl BGM were added carefully. Parallel cultures were performed with the same HFBSCs cells of the co-culture, in order to establish immunophenotypic changes of the cells. Every other day, half of the medium was refreshed. After 7 days in culture, the explants were taken out of the culture and fixed with pre-warmed (37°C) 1% formaldehyde in PBS. To protect copGFP from photobleaching, the explants were stored in 1.5 ml amber-colored Eppendorf^®^ Safe-Lock microcentrifuge tubes (Sigma-Aldrich) at 4°C.

### Immunohistochemistry

The fixed quarter-turn explants and control cells were washed with phosphate-buffered saline (PBS) containing 0.05% Tween-20 (Promega, Madison, WI, USA) for 5 min and permeabilized with 0.3% Triton X-100 (Sigma-Aldrich) in PBS containing 5% normal goat serum (Dako, Glostrup, Denmark) for 60 minutes. After washing with PBS/Tween-20, the specimens were incubated in blocking solution containing 0.1% Triton X-100 and 5% normal goat serum in PBS for 30 minutes. The specimens were incubated overnight with primary antibodies at room temperature. Primary antibodies used in this study are listed in Table 1. After washing, the specimens were incubated in blocking solution for 10 minutes followed by incubation with goat anti-mouse or goat anti-rabbit fluorochrome-conjugated (Alexa® Fluor 555, 1:300; Alexa® Fluor 750, 1:50) secondary antibodies, at room temperature for 90 minutes. Nuclei were counterstained with DAPI (Invitrogen) at a dilution of 1:1000 in PBS for 15 minutes. The specimens were mounted in a drop of Roti®-Mount FluorCare (Carl Roth GmbH + Co. KG, Karlsruhe, Germany) under a cover glass. All specimens were examined with a Leica DM5500 B fluorescence microscope (filter settings: TXR, Cy7, FITC and DAPI), equipped with a Leica DFC365 FX digital camera. Digital images were acquired and stored using Leica Application Suite X (LAS X) software. All images were subsequently processed using Adobe® Photoshop® CC software (version 2014.2.1).

**Table 1:** Primary antibodies used for immunohistochemistry (in alphabetical order).

Antibody	Dilution	Manufacturer	Catalogue Number	Positive control	Marker specific for
**ATOH1**	1:100	Acris	AP00308PU-N	Developing hair cells	neurons, hair cells and supporting cells in the developing cochlea
**TUBB3**	1:100	Abcam	AB78078	Mouse sciatic nerve	developing and young neurons
**GFAP**	1:100	Abcam	AB10062	Human astrocytes	glial cells
**KROX-20**	1:100	Covance	PRB-236P	RT4-D6P2T cells	myelinating Schwann cells
**MPZ**	1:100	Prof. Dies Meijer		RT4-D6P2T cells	myelinating Schwann cells, myelinated spiral ganglion neurons
**Myosin VIIa**	1:1000	Proteus	25–6790	Mouse organ of Corti	adult hair cells
**Nestin**	1:200	Biosensis	M-1385-100	C17.2 neural stem cells	neural crest cells and neural progenitors
**NF-H**	1:100	Abcam	AB8135	Mouse brain	mature neurons
**NF-M**	1:100	DSHB	2H3	Mouse brain	mature neurons
**S100**	1:100	DAKO	ZO311	RT4-D6P2T cells	Schwann cells
**SOX2**	1:1000	Abcam	AB97959	M14 melanoma cells	developing hair cells
**SOX9**	1:500	Millipore	AB5535	M14 melanoma cells	neural crest stem cells

### Phalloidin staining

The presence of F-actin in quarter-turn explants was demonstrated with Alexa® Fluor 555-conjugated phalloidin (Invitrogen, Carlsbad, CA, USA). F-actin is a cytoskeleton marker and used to stain extant cells in the quarter-turn explant. Fixed specimens were washed three times with PBS and incubated with phalloidin (diluted in PBS containing 0.05% Tween-20) for 60 minutes followed by three washing steps with PBS. Next, nuclei of the specimens were counterstained with DAPI (diluted 1:1000 in PBS) for 15 minutes followed by washing with PBS and covered with Roti®-Mount FluorCare and a cover glass.

### Statistical analyses

Data of the migration experiment were processed in Microsoft Excel 2010 (Microsoft Corporation, Redmond, WA, USA) and expressed as mean ± SD. Statistically analysis was performed using GraphPad Prism version 6.02 software (GraphPad Software, La Jolla, CA, USA).

## Results

### Adult hair cells degenerate in BGM within 2 hours

Quarter-turn explants, which were fixed in formaldehyde immediately after dissection, stain for the adult hair cell marker myosin VIIa (n = 6, Fig 1A). When cultured in BGM for 2 hours–not in artificial perilymph–only some diffuse, pectinate staining for myosin VIIa could be observed, indicating that already most hair cells had been lost during such a short period of culture (n = 3, Fig 1B). After incubation in BGM for 36 hours, the specimens were completely devoid of staining for myosin VIIa (n = 3, Fig 1C).

### Transduced HFBSCs migrate towards modiolus explant

EF1α-copGFP HFBSCs crossed the cell-free zone (radius: 7.5 ± 0.6 mm μm) with a velocity of 80.5 ± 6.1 μm/h (n = 4) towards the modiolus explant and arrived, often in groups, at the explant within 2–3 days (Fig 2C and 2C’). Cells located underneath the explant could clearly be distinguished from the modiolus tissue by adjusting the focus level during observation, i.e., to verify whether fluorescent cells arrived at the tissue (Fig 2D). In control experiments, i.e. wells without a modiolus explant in the center, cells proliferated at the border of the well, but did not migrate towards the center of the well (data not shown).

### Stem cells form a distinct fascicular pattern

In all cultures of the migration experiment, fluorescent HFBSCs invaded the explants within 2 days (2.2 ± 1.6 days) after arrival. Within the next 5 days (5.2 ± 1.6 days), the cells formed a distinct fascicular pattern (n = 10). To shorten culture time, the protocol was adapted by seeding 2x10^4^ EF1α-copGFP HFBSCs directly onto quarter-turn explants (n = 50). We did not observe differences in fascicular organization of HFBSCs in the explants between both approaches.

**Fig 3:**
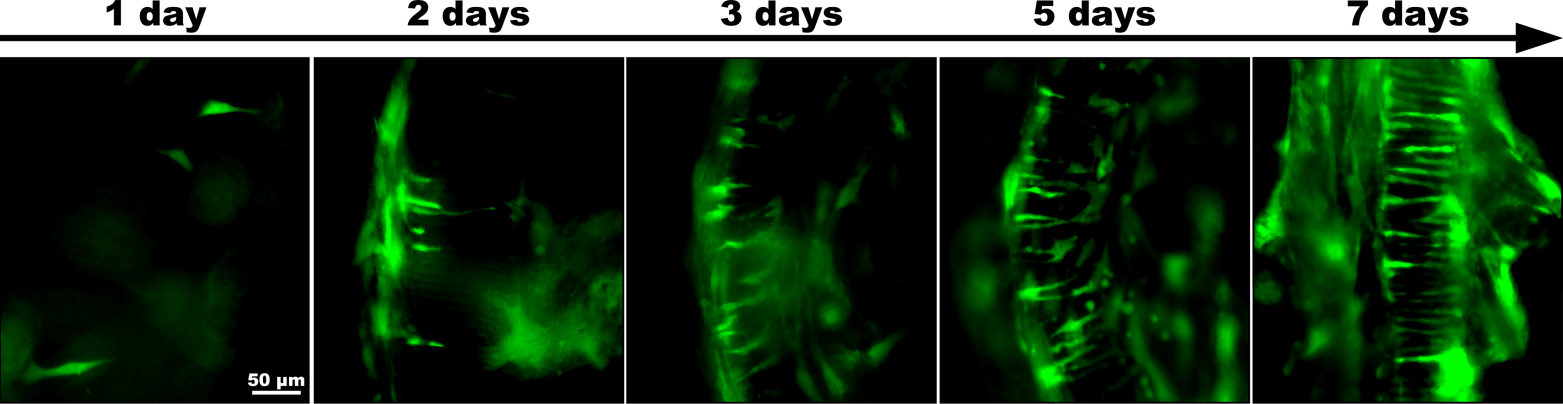
Distinct fascicular pattern visible within 5 days. One day after seeding, fluorescent HFBSCs migrated under the quarter-turn explant and began migrating into the explant within the next day (2 days). Over time, more EF1α-copGFP HFBSCs settled in the explant (3 days) forming a distinct fascicular pattern after 5 days. Within the next two days the pattern enhanced as the number of HFBSCs within the explant increased.

The time course of the fascicular pattern formation by HFBSCs is depicted in Fig 3. At day 1, cells are visible underneath the tissue, attached to the bottom of the well and invaded the explant after 2 days of co-culture. At day 3, the number of HFBSCs increased within the tissue and cells formed a distinct fascicular pattern within 5 days. This pattern became more enhanced over the next two days, up to day 7.

### Similar distinct fascicular pattern formation of HFBSCs transduced with EF1α-copGFP and DCX-copGFP

**Fig 4:**
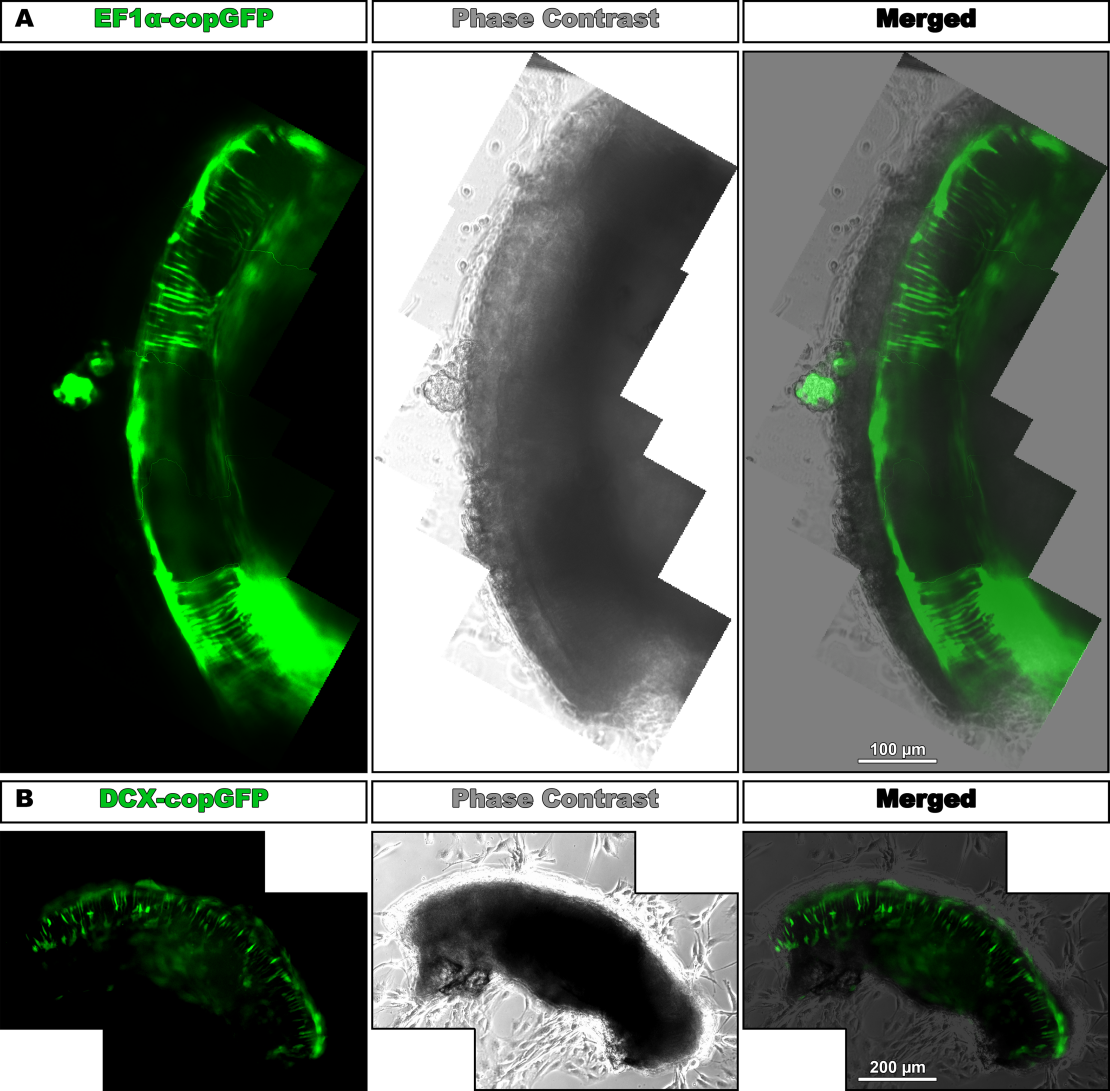
EF1α-copGFP HFBSCs and DCX-copGFP HFBSCs form a similar distinct fascicular pattern. (A) Set of stitched fluorescence images showing the distinct fascicular pattern that is formed by the copGFP-expressing cells (green). The stitched phase-contrast images depict the morphology of the quarter-turn explant (gray) and the merged set of images reveals the position of the green fluorescent cells within the explant. (B) DCX-copGFP cells formed a similar fascicular pattern of fluorescent cells within the explant (green). The merged image of phase-contrast (gray) and fluorescence images depicts the location of DCX-copGFP-expressing cells within the explant.

After obtaining a fascicular pattern of EF1α-copGFP HFBSCs in quarter-turn explants of adult mice (Fig 4A), we investigated the role of the neuronal migration protein DCX in the process of pattern formation and seeded DCX-copGFP HFBSCs onto quarter-turn explants. During the next 4 days (4.1 ± 0.9 days), different cells became fluorescent and at day 5 (5.3 ± 0.9 days after seeding; n = 7), the fluorescent HFBSCs were organized in a distinct fascicular pattern (Fig 4B), similar to the pattern displayed by EF1α-copGFP HFBSCs. Therefore, the formation of a fascicular pattern is not related to the use of a specific promoter (p = 0.862, two-tailed, unpaired *t*-test, 95% confidence interval).

### Fluorescent EF1α-copGFP HFBSCs, in the fascicular pattern, immunostain for (auditory) neuronal markers

**Fig 5:**
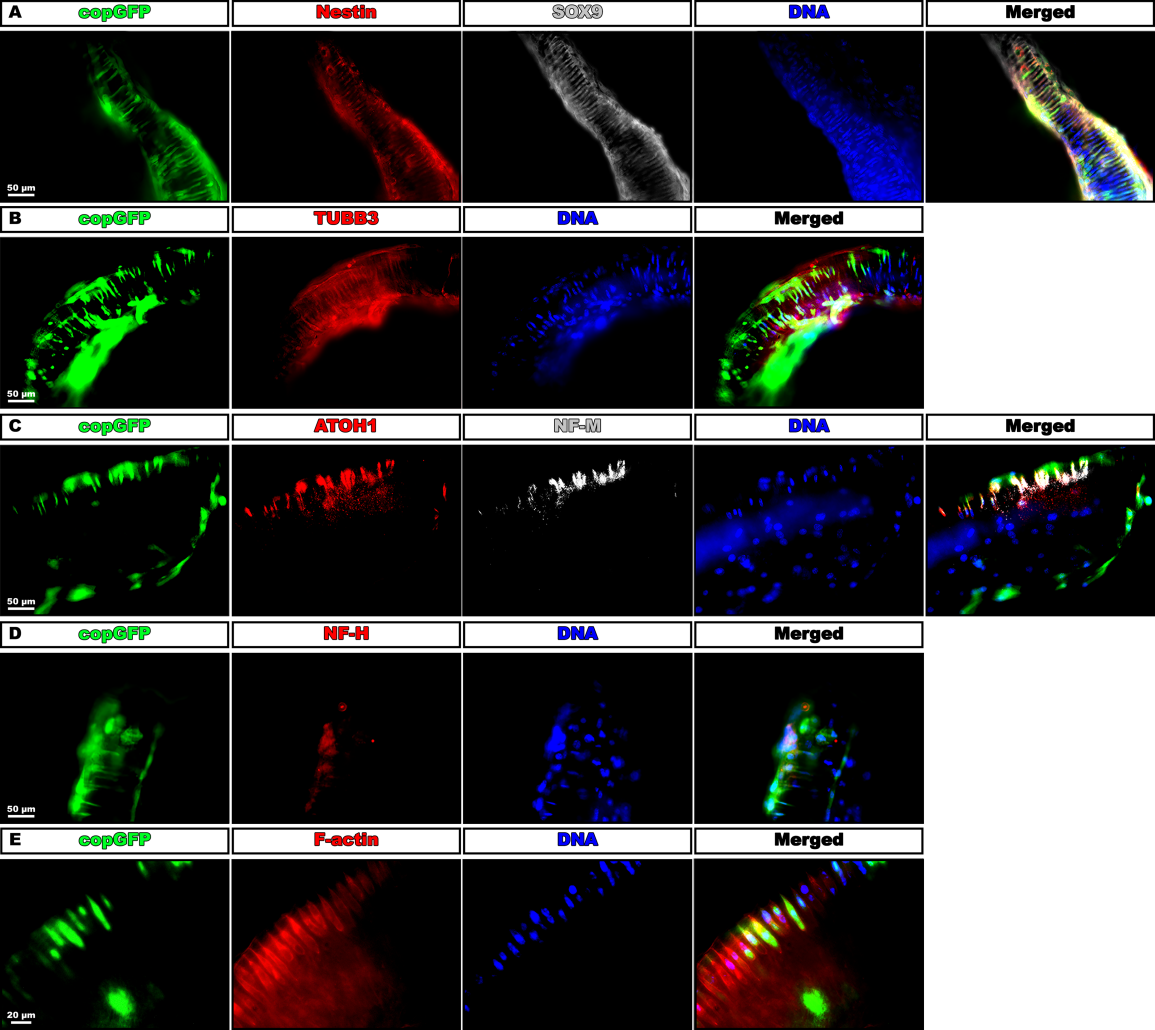
HFBSCs within quarter-turn explants stain for nestin, SOX9, TUBB3, NF-M, ATOH1 and F-actin. (A) Cop-GFP-expressing cells (green) in the explants stain for nestin (red) and SOX9 (gray). Nuclear DNA is counterstained with DAPI (blue). Merged images show co-localization of nestin and SOX9 in copGFP-expressing cells. (B) All green-fluorescent cells and some non-fluorescent cells co-localize with TUBB3 (red) in the explants. (C) Most copGFP-expressing cells (green) stain positive for ATOH1 (red) and NF-M (gray) was exclusively positive in fluorescent HFBSCs. (D) NF-H (red) was negative in co-cultured HFBSCs. (E) In the same row of cells, some transduced HFBSCs (green) were positive for F-actin (red) within the explant. In the explant also many non-fluorescent F-actin-positive cells are present.

Fluorescent (copGFP-containing) cells in the modiolus explants showed immunostaining for the NCSC marker SOX9 (Fig 5A). Nestin, a marker for NCSCs and neural progenitors, was weakly positive in most copGFP-expressing and SOX9-positive HFBSCs (Fig 5A). TUBB3, a marker for young neurons, was weakly positive, not only in fluorescent HFBSCs but also in some non-fluorescent (endogenous) cells (Fig 5B). Medium-chain neurofilament protein (NF-M), generally expressed early during neuronal maturation, was exclusively positive in fluorescent HFBSCs (Fig 5C), while the marker for mature neurons, heavy-chain neurofilament protein (NF-H), was negative (Fig 5D). Most copGFP-positive cells arranged in the fascicular pattern immunostained for ATOH1, a marker for neurons and hair cells in the developing cochlea (Fig 5C). The majority of ATOH1-positive cells was also positive for NF-M. Since hair cells are not present in the explant, the single ATOH1-positive, copGFP-negative cell is most likely a HFBSC that has not been successfully transduced (transduction efficiency is ± 84%). The immunostaining for SOX2, a marker for developing hair cells, was negative. In the pattern, some copGFP-positive HFBSCs, but also many non-fluorescent cells, were immunopositive for F-actin (Fig 5E). The non-GFP-fluorescent, F-actin-positive cells demonstrate a morphology reminiscent of interdental cells [19].

Immunostaining for the adult hair cell marker myosin VIIa was negative, both in the hair cell survival experiment (Fig 1) and in the co-cultures, indicating that the outer and inner hair cells were lost from the explants, even in short-term cultures. None of the glial markers (GFAP, S100, KROX-20 and MPZ) were immunopositive in copGFP-positive HFBSCs. The immunoprofile of the HFBSCs in the pattern compared to non-co-cultured HFBSCs are listed in Table 2. This table shows that HFBSCs changed their phenotype after incorporation in the explants and that they became positive for several (young) neuronal markers and negative for all glial markers. All pertinent positive and negative controls showed appropriate results.

**Table 2:** Immunoprofile of the HFBSCs in the pattern as compared to non-co-cultured HFBSCs.

	Antibody	HFBSCs	HFBSCs in pattern
**Neural Crest**	SOX9	weakly positive	positive
	Nestin	positive	weakly positive
**Neuron**	TUBB3	negative	weakly positive
	NF-M	weakly positive	positive
	NF-H	negative	negative
**Hair Cell**	ATOH1	weakly positive	positive
	SOX2	weakly positive	negative
	Myo VIIa	negative	negative
**Glial Cell**	GFAP	negative	negative
	S100	negative	negative
	KROX20	negative	negative
	MPZ	negative	negative

Antibodies are sorted by group (NCSC, neuron, hair cell, glial cell). Positive staining is indicated by a dark gray background and weakly positive staining is in light gray, while negative staining is white, according to the respective text.

## Discussion

The results show that our population of HFBSCs meets three important requirements to enable their contribution to successful inner ear regeneration: they are migratory, they integrate into cochlear tissue, and have the capability to adapt a neuronal phenotype depending on the microenvironment.

The cell migration velocity of HFBSCs was relatively fast (80.5 ± 6.1 μm/h) as compared to other cell types as determined in wound-healing migration assays [20–23], indicating that they conserved the migratory nature of NCSCs. Migration of cells is to a large extent dependent on the type of extracellular matrix (ECM) and the presence of chemical attractants, such as growth factors. The cells in this experiment were seeded on a poly-D-lysine matrix, which allows migration of neural crest descendants [24, 25]. On the other hand, we considered it unlikely that chemotactic factors were present in the tissue, because the cultured explants were devoid of hair cells. However, the remaining non-sensory cells may have expressed growth factors, which could function as a chemoattractant for HFBSCs [26]. In addition, it is generally known that certain growth factors can directly bind to ECM proteins [27]. Hence, it is possible that neural growth factors are present in the ECM of the explants, i.e. in the spiral limbus, the osseous spiral lamina and the basilar membrane. In the co-culture experiment, growth factors from the non-sensory cells and the ECM may therefore have attracted the HFBSCs.

Within five days upon arrival into the explant, the HFBSCs formed a distinctly fascicular pattern while co-localizing with F-actin-positive, non-GFP-fluorescent cells (Fig 3E). Within seven days after their settlement, HFBSCs developed a neuronal phenotype corresponding to the expression of copGFP under regulation of the DCX promoter. This is an unusually rapid neuronal differentiation, for it has been reported that neuronal progenitors generally need three weeks to achieve NF-M positivity [28]. We hypothesize that there are several biochemical cues present in the explants, probably working in synergy, promoting a neuronal phenotype of the locally present HFBSCs. Firstly, although hair cells have disappeared, neural growth factors could still be present in supporting cells or be bound to ECM proteins. Secondly, the type of ECM proteins may be of importance: among the different classes of ECM molecules that are present in the cochlea, laminin has been shown to be particularly effective in stimulating neuronal differentiation [25, 29–31]. Thirdly, given the fasciscular pattern of HFBSCs within the explants, the cells are probably dwelling in or near Huschke’s teeth, i.e, in the upper region of the spiral limbus. This seems a plausible assumption, for the non-copGFP-fluorescent cells near the F-actin-positive, copGFP-fluorescent HFBSCs (Fig 3E) are morphologically similar to interdental cells. It is known that a columnar-shaped ECM stimulates neuronal differentiation of cells [32, 33]. Hence, the columnar plates of the spiral limbus matrix (Huschke’s teeth) [34] may well have stimulated HFBSCs to differentiate into a neuronal phenotype.

In our opinion, our experiments show that HFBSCs are tolerated in inner ear tissue from adult animals. Indeed, HFBSCs do intermingle with extant cells of the modiolus and can subsequently adapt to the microenvironment. Furthermore, in spite of the absence of trophic factors from hair cells, HFBSCs changed their phenotype into that of young neuronal cells (Fig 5). Various studies have shown that stems cells isolated from the bulge area of mouse and human hair follicles express nestin, a marker for NCSCs and neural progenitors [35–45]. In these reports, it was established that nestin-positive HFBSCs can differentiate towards a variety of cells such as neurons, glia cells, melanocytes, keratinocytes as well as smooth- and cardiac muscle cells. This broad differentiation capacity illustrates the potential of HFBSCs to serve many regenerative purposes. Our finding that nestin-positive HFBSCs differentiate towards a neuronal lineage, corroborates the pertinent above-mentioned reports (Table 2).

Hence, our report presents clear evidence that these cells may be good candidates for *in vivo* regenerative experiments in an animal model of SNHL. In upcoming studies we will use HFBSCs in CI-implanted guinea pigs, deafened by means of concomitant administration of kanamycin and furosemide. In this model no hair cells are present, similar to deaf patients and to our *in vitro* experiments.

## Conclusion

In conclusion, the present observations show that HFBSCs are migratory and can successfully integrate into inner ear tissue, i.e., into the modiolus, while adapting a neuronal phenotype. Hence, the results of this study show the potential of HFBSCs for auditory nerve repair. Our next step will be to investigate *in vivo* if HFBSCs after transplantation into the spiral ganglion receive the appropriate biochemical stimuli to differentiate into auditory neurons and/or glial cells.
